# Identification of a genetic locus for autosomal dominant infantile cataract on chromosome 20p12.1-p11.23 in a Chinese family

**Published:** 2008-10-22

**Authors:** Shirong Zhang, Mugen Liu, Jia Mei Dong, Ke Yin, Pengyun Wang, Juan Bu, Jing Li, Yan Sheng Hao, Ping Hao, Qing Kenneth Wang, Lejin Wang

**Affiliations:** 1 Key Laboratory of Molecular Biophysics of Ministry of Education, College of life Science and Technology, Wuhan, P.R. China; 2 Center for Human Genome Research, Huazhong University of Science and Technology, Wuhan, P.R. China; 3 Department of Pediatric Ophthalmology & Strabismus, Peking University Eye Center and Peking University Third Hospital, Beijing, P.R. China; 4 Guiyang College of Traditional Chinese Medicine, Guiyang, P.R. China; 5 Shangqiu Eye Hospital, Henan, P.R. China; 6 Center for Cardiovascular Genetics, Department of Molecular Cardiology, Lerner Research Institute, Cleveland Clinic, Cleveland, OH

## Abstract

**Purpose:**

To map a gene responsible for infantile cataract in a large four-generation, non-consanguineous Chinese family.

**Methods:**

Twenty-two family members including 17 cataract patients in the Chinese family were analyzed clinically. All family members were genotyped with 382 microsatellite markers that provide genome-wide coverage every 10 cM. Linkage analysis was performed to identify the chromosomal location of the infantile cataract gene in the family. Candidate genes were studied by direct DNA sequence analysis.

**Results:**

Genome-wide linkage analysis provided evidence for a genetic locus for infantile cataract on chromosome 20p12.2-20p11.23. The maximum LOD score was 5.15 for marker D20S471 at a recombination fraction of 0. Fine mapping defined the cataract gene within a 7.4 Mb interval between markers D20S915 and D20S912. No mutation was detected in potential candidate genes, *BFSP1* and *CHMP4B*.

**Conclusions:**

Our results suggest that there is a new gene for infantile cataract on chromosome 20p12.2-p11.23. Our results suggest that new genes for infantile cataract could be found through further study of candidate genes at the 20q locus, which may provide insights into the pathogenic mechanisms of cataracts.

## Introduction

Congenital cataract is a significant cause of hereditary visual impairment in childhood. Its incidence is estimated to be 2.2-2.49 per 10,000 live births and may account for one-third of total blindness in infants [1-3]. Congenital cataract is genetically and clinically highly heterogeneous, and various inheritance patterns have been reported including autosomal dominant, autosomal recessive, and X-linked forms [4,5]. To date, 21 genetic loci have been identified, and 16 causative genes have been identified for autosomal dominant congenital cataract (ADCC), the most common familial form [6]. The phenotype of ADCC also varies markedly in morphology, affecting the nuclear, cortical, polar, and other sections of the lens [7]. Total cataract is characterized by opacity of all lens fibers. For autosomal dominant total cataract, one mutation in the heat shock transcription factor (*HSF4*) gene was reported by us [8], and another mutation was identified in a gene encoding the major intrinsic protein (MIP, MIP26) of the lens [9]. For autosomal recessive total cataract, a mutation was reported in the gene encoding the α-A component of α-crystallin (*CRYAA*) [10].

There are three ADCC families linked to chromosome 20 including a Japanese autosomal dominant posterior cataract family linked to chromosome 20p12–20q12 [11] and a Chinese family with autosomal dominant progressive congenital zonular nuclear cataract that is linked to chromosome 20p12.2-20p11.23 [12]. Very recently, a white (U.S.A.) family with progressive childhood posterior subcapsular cataracts has been reported to be linked to 20q, and *CHMP4B* was identified as the pathogenic gene for ADCC at this locus [13].

In this study, we analyzed a four-generation, non-consanguineous Chinese family diagnosed as having infantile total cataract. It is different from other congenital cataract because there is no amblyopia presented posteriorly with intraocular lens transplantation. Because the cataract was present at the age of 10–12 years and showed progressive development of lens opacities within one to two years and decreased visual acuity. Our genome-wide linkage screen mapped the new infantile cataract pathogenic gene on chromosome 20p12.2-20p11.23. The maximum LOD score reached 5.15 for marker D20S471 at a recombination fraction of 0. The infantile cataract gene was further defined within a 12.5 cM (7.5 Mb) interval between markers D20S915 and D20S912. In this region, the potential candidate genes include *BFSP1* and the newly-reported pathogenic gene, *CHMP4B*. However, direct DNA sequence analysis did not identify any mutation in the two candidate genes. Our results suggest that a gene for infantile total cataract is located on chromosome 20p11.23.

## Methods

### Study participants

**Figure 1:**
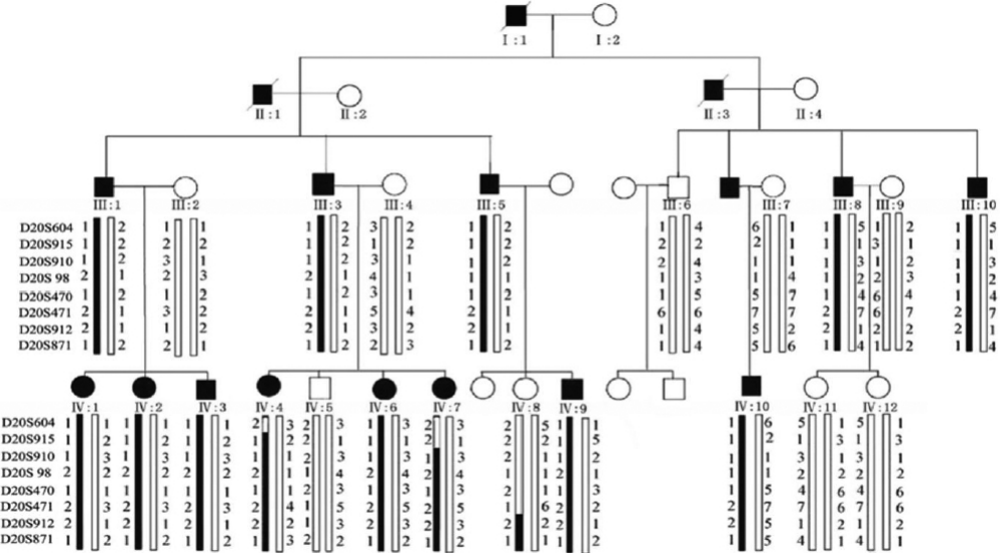
Pedigree structure of a Chinese family with autosomal dominant total cataract. The genotyping results are shown for markers D20S604, D20S915, D20S910, D20S98, D20S470, D20S471, D20S912, and D20S871. The haplotype inherited from the affected parents is shown on the left of each pair. The vertical dark-box bars represent the mutant haplotype, and the open bars are normal haplotypes.

We recruited a four-generation, non-consanguineous Chinese family from the Henan province with multiple family members diagnosed as having total cataract. The family displayed an autosomal dominant inheritance pattern (Figure 1). There are 17 patients. Blood samples were obtained from 14 living patients.

**Figure 2:**
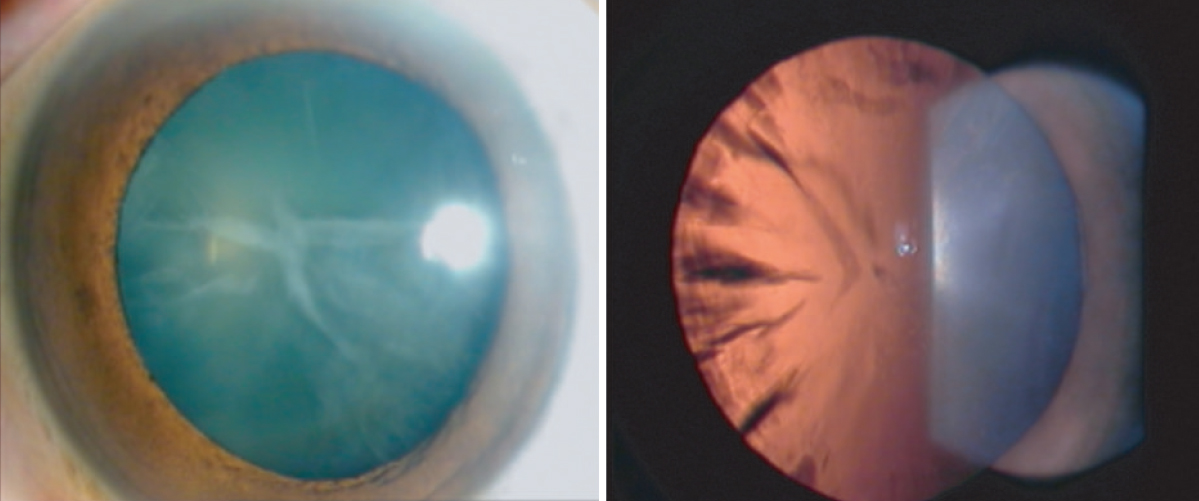
Slit-lamp image of the lens opacity from an individual with congenital total cataract from the Chinese family. A pre-operative photo of the left eye from patient III:1 (see Figure 1) illustrates the typical cataract identified in this pedigree. The cataract was present around 10-12 years of age and showed progressive development of lens opacities within one to two years and decreased visual acuity, but it is different from others because no amblyopia is presented posteriorly with IOL transplantation. Slit lamp examinations were performed to characterize the lens phenotype. The phenotype is lamellar cataract. Perinuclear-shaped total opacities were restricted to the lamellae and nucleus. The total cataract morphology was identified for all affected members in the family (data not shown).

Complete medical history analyses and ophthalmic examinations were performed on all living family members. The examinations include assessment of visual acuity and a detailed examination of the ocular lens under a slit lamp to determine the disease status. The results are shown in Table 1. In this family, the phenotype of lens opacity showed total cataract in morphology (Figure 2). The initial clinical manifestation for all affected members was opacity of the lens and decreased visual acuity at the age of 10–12 years. Interestingly, the cataract phenotype starts to manifest as early as just after birth (Figure 2).

**Table 1:** Clinical characteristics of family members at risk for congenital total cataract in the Chinese family.

**Pedigree number**	**Sex/Age**	**Clinical disease**	**IOL transplantation**	**Visual acuity OD**	**Visual acuity OS**
III:1	M/53	Y	Y	20/40	20/60
III:2	F/52	N	N	20/20	20/20
III:3	M/51	Y	Y	20/50	20/50
III:4	F/49	N	N	20/20	20/20
III:5	M/49	Y	Y	20/40	20/30
III:6	M/53	N	N	20/20	20/20
III:7	F/48	N	N	20/20	20/20
III:8	M/44	Y	Y	20/60	20/40
III:9	F/42	N	N	20/30	20/20
III:10	M/38	Y	Y	20/20	20/20
IV:1	F/28	Y	Y	20/20	20/20
IV:2	F/26	Y	Y	20/30	20/20
IV:3	M/22	Y	Y	20/20	20/20
IV:4	F/25	Y	Y	20/30	20/20
IV:5	M/24	N	N	20/20	20/20
IV:6	F/21	Y	Y	20/30	20/30
IV:7	F/20	Y	Y	20/20	20/20
IV:8	F/9	N	N	20/20	20/20
IV:9	M/7	Y	Y	20/20	20/20
IV:10	M/20	Y	Y	20–40	20/40
IV:11	F/18	N	N	20/20	20/20
IV:12	F/16	N	N	20/20	20/20

Y: affected and with IOL transplantation; N: unaffected and without IOL transplantation. OD = right eye; OS = left eye.

Informed consent was obtained from all participants, and the study was in accordance with the tenets of the Declaration of Helsinki on human subject research.

### Genotyping

Genomic DNA was extracted from the peripheral blood of 22 family members with a DNA isolation kit for mammalian blood (Wizard Genomic DNA Purification kit; Promega, Madison, WI). The DNA samples were quantified by a spectrophotometer and diluted to 25 ng/μl for polymerase chain reaction (PCR) amplification.

The initial genome-wide screen was performed with 382 highly polymorphic fluorescent markers (PRISM Linkage Mapping Set MD-10, Applied Biosystems, Foster City, CA) that have an average spacing interval of 10 cM over the entire human genome. Other fluorescently labeled markers were selected and designed according to the Marshfield Clinic Medical Genetics database for fine mapping. Each PCR genotyping reaction was performed in a 5 μl volume containing 50 ng of genomic DNA, 10 μM dye-labeled primer pairs, 0.5 μl 10X PCR buffer (GeneAmp Buffer II, Applied Biosystems), 0.5 μl 10 mM dNTP mix, and 0.2 U of Taq DNA polymerase (AmpliTaq Gold Enzyme, Applied Biosystems). Amplification was performed in a GeneAmp 9700 PCR system (Applied Biosystems). PCR products (1 μl) from each DNA sample was pooled and mixed with 0.2 μl of Liz Size Standard-500 and 9 μl of Hi-Di formamide (both from Applied Biosystems, Foster City, CA). The mixture was then fractionated by electrophoresis and visualized on a 3100 Genetic Analyzer (Applied Biosystems). The GenScan 3.1 and GeneMapper 2.5 software (Applied Biosystems) were used to analyze the alleles.

### Linkage analysis

Two-point linkage analysis was performed using the MLINK program from the LINKAGE program package (version 5.2). The linkage was performed assuming an autosomal dominant inheritance pattern, 99% penetrance, and a disease gene frequency of 0.0001.

Multipoint linkage analysis was computed using GENEHUNTER version 2.1 running on the Linux operating system, and the marker order and positions were based on the Marshfield Clinic Medical Genetics map (Table 2). Haplotype analysis was performed using the Cyrillic 2.1 (Cyrillic Software, Setauket, NY) program and manual prediction.

**Table 2:** Two-point LOD scores from linkage analysis of total cataract for markers on chromosome 20p12–20q12 in the Chinese family.

**Marker**	**Position (cM)**	**LOD score at θ**								**Z_max_**	**θ_max_**
		**0.0**	**0.05**	**0.1**	**0.15**	**0.2**	**0.25**	**0.3**	**0.4**		
D20S604	39.94	−2.50	1.10	1.39	1.42	1.33	1.17	0.97	0.49	1.43	0.15
D20S915	34.22	2.73	2.44	2.15	1.85	1.54	1.22	0.89	0.28	2.73	0.0
D20S910	35.51	3.78	3.51	3.18	2.81	2.40	1.97	1.51	0.56	3.78	0.0
D20S98	37.65	4.02	3.65	3.25	2.84	2.41	1.97	1.51	0.60	4.02	0.0
D20S470	39.25	3.76	3.45	3.11	2.75	2.38	1.99	1.60	0.78	3.76	0.0
D20S471	42.28	5.15	4.7	4.23	3.74	3.23	2.69	2.12	0.95	5.15	0.0
D20S912	46.71	−2.91	0.38	0.63	0.67	0.61	0.49	0.34	0.06	0.67	0.15
D20S871	48.61	0.58	0.82	0.88	0.86	0.79	0.69	0.56	0.27	0.88	0.1

### Mutational analysis

All exons and exon-intron boundaries of candidate genes, *BFSP1* and *CHMP4B*, were amplified by PCR, purified, and sequenced using the BigDye Terminator Cycle Sequencing v3.1 kit (Applied Biosystems).

## Results

**Figure 3:**
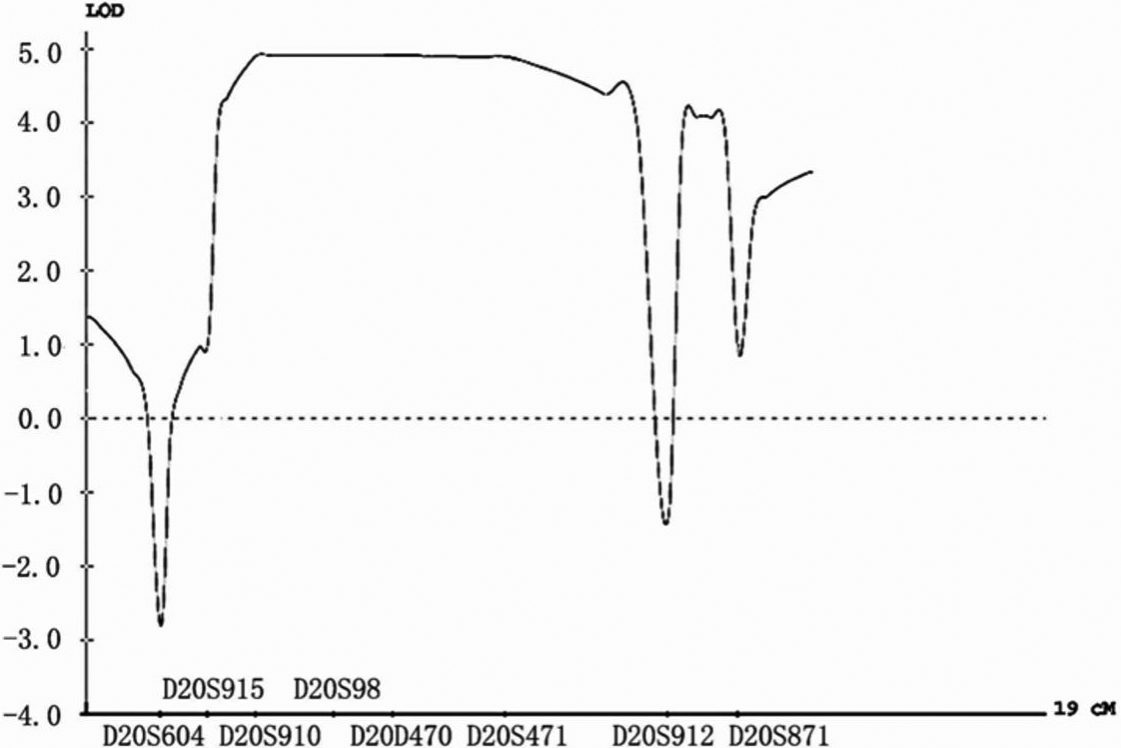
Multipoint linkage analysis spanning 19 cM between total cataract phenotype and markers from D20S604 to D20S871 using the GENEHUNTER 2.1 program. Genetic distance between the markers is as indicated in Table 1.

We clinically characterized a large Chinese family with a diagnosis of total cataract, which manifests as early as at birth (infantile cataract, Figure 2). A genome-wide genotyping scan was performed for 22 members of the family including 14 living patients, five normal individuals, and three married spouses, using 382 microsatellite markers covering the entire human autosome every 10 cM. We obtained a positive two-point LOD score of 2.73 at θ=0 with marker D20S195 (Table 2). The two markers flanking D20S195 were uninformative. Fine mapping was then carried out with markers near D20S195, and multiple markers showed LOD scores greater than 3.0 (Table 2). Marker D20S471 showed the highest LOD score of 5.15. Multipoint linkage analysis showed a peak LOD score of nearly 5.0 for the chromosomal region from D20S910 to D20S471 (Figure 3). These results revealed that the gene for infantile total cataract in the Chinese family is linked to markers D20S910 to D20S471 on chromosome 20 with significant LOD scores.

Haplotype analysis was constructed for eight markers on chromosome 20p12.2-p11.23. Obligate recombination events were identified in patient IV-4 between D20S604 and D20S915 and in individual IV-7 between D20S915 and D20S910. Thus, D20S915 was defined as the left flanking marker for the locus. One non-obligate recombination event was detected in a normal individual, IV-8, between D20S471 and D20S912, which defines D20S912 as the right flanking marker for the locus.

Mutational analysis of two candidate genes on chromosome 20, *BFSP1* and *CHMP4B*, did not reveal any disease-associated mutation.

## Discussion

Using genome-wide genetic linkage analysis, we have shown that a gene for an autosomal dominant infantile total cataract is located on the short arm of chromosome 20, within a 7.4 Mb interval between markers D20S915 and D20S912. Recently, Shiels at el. [13] reported linkage of autosomal dominant progressive childhood posterior sub-capsular cataracts to chromosome 20q in a Caucasian family and identified the pathogenic gene as *CHMP4B* in a refined disease interval of 3.5 cM. We used direct DNA sequence analysis for mutational analysis of *CHMP4B*, but no mutation was identified. Furthermore, *CHMP4B* is located outside of the refined disease locus. Thus, the infantile cataract gene on chromosome 20p12.2-p11.23 in the Chinese family is not *CHMP4B*.

One Japanese autosomal dominant posterior cataract family has been linked to chromosome 20p12–20q12 [11]. A Chinese autosomal dominant progressive congenital zonular nuclear cataract family has been linked to chromosome 20p12.2-p11.23 [12]. Interestingly, the Chinese family with infantile total cataract under this study is also linked to the same region. It is possible that the same gene is responsible for three different types of cataracts in the Japanese family, the Chinese family, and the family under this study. Our infantile total cataract locus is 2 cM smaller than the progressive congenital zonular nuclear cataract locus identified in the other Chinese family and much smaller than the posterior cataract locus identified in the Japanese family. On the other hand, due to the highly clinical and genetic heterogeneity of congenital cataracts, it may be possible that the distinct genes contributed to the different cataract phenotype at this specific chromosomal region. Future identification of these specific genes should be able to distinguish the two hypotheses.

There are no other obvious candidate genes for autosomal dominant total cataract on chromosome 20p12.2-p11.23 except for *BSFP1*. *BSFP1* encodes the beaded filament structural protein 1, a lens-specific intermediate filament-like protein, which functions as a major cytoskeletal element of the eye lens and is essential to the optical properties of eye lens. Mutations in *BFSP2* have been reported to be associated with autosomal dominant congenital cataract [14,15]. In a consanguineous family of Indian origin with autosomal recessive juvenile onset cortical cataract, linkage was detected with markers between D20S852 and D20S912 (peak LOD=5.4 with D20S860), and one homozygous deletion in *BFSP1* was identified [16]. Heterozygous carriers did not develop cataracts. Direct DNA sequence analysis of the entire coding region and exon-intron boundaries of *BFSP1* has been previously conducted in the Japanese and Chinese cataract families linked to chromosome 20, but no mutation was identified [11,12]. We also performed direct DNA sequence analysis of *BFSP1* for the proband from the family under this study but did not detect any mutation. Although we cannot exclude the possibility that a *BFSP1* mutation in the promoter or an intron may be associated with cataract in the family, this is unlikely because our results are consistent with the findings by Yamada et al. [11], Li et al. [12], and Ramachandran et al. [16] that heterozygous carriers for a *BFSP1* deletion were phenotypically normal.

In summary, these results indicate that there is new gene on chromosome 20p12.2-p11.23 that is responsible for infantile total cataract. The disease gene interval has been defined between markers D20S915 and D20S912, a 7.4 Mb region. Future studies of the candidate genes within the locus should identify the specific gene, which will provide further important insights into the genetic basis of infantile cataracts.
